# Functional implications of corticosteroid-binding globulin *N*-glycosylation

**DOI:** 10.1530/JME-17-0234

**Published:** 2017-12-22

**Authors:** Marc Simard, Caroline Underhill, Geoffrey L Hammond

**Affiliations:** Department of Cellular and Physiological Sciences The University of British Columbia, Vancouver, British Columbia, Canada

**Keywords:** SERPIN, glycoprotein, cortisol, protein structure/function, protease

## Abstract

Corticosteroid-binding globulin (CBG) is a plasma carrier of glucocorticoids. Human and rat CBGs have six *N*-glycosylation sites. Glycosylation of human CBG influences its steroid-binding activity, and there are *N*-glycosylation sites in the reactive center loops (RCLs) of human and rat CBGs. Proteolysis of the RCL of human CBG causes a structural change that disrupts steroid binding. We now show that mutations of conserved *N*-glycosylation sites at N238 in human CBG and N230 in rat CBG disrupt steroid binding. Inhibiting glycosylation by tunicamycin also markedly reduced human and rat CBG steroid-binding activities. Deglycosylation of fully glycosylated human CBG or human CBG with only one *N*-glycan at N238 with Endo H-reduced steroid-binding affinity, while PNGase F-mediated deglycosylation does not, indicating that steroid binding is preserved by deamidation of N238 when its *N*-glycan is removed. When expressed in *N*-acetylglucosaminyltransferase-I-deficient Lec1 cells, human and rat CBGs, and a human CBG mutant with only one glycosylation site at N238, have higher (2–4 fold) steroid-binding affinities than when produced by sialylation-deficient Lec2 cells or glycosylation-competent CHO-S cells. Thus, the presence and composition of an *N*-glycan in this conserved position both appear to influence the steroid binding of CBG. We also demonstrate that neutrophil elastase cleaves the RCL of human CBG and reduces its steroid-binding capacity more efficiently than does chymotrypsin or the *Pseudomonas aeruginosa* protease LasB. Moreover, while glycosylation of N347 in the RCL limits these activities, *N*-glycans at other sites also appear to protect CBG from neutrophil elastase or chymotrypsin.

## Introduction

Corticosteroid-binding globulin (CBG) is a plasma glycoprotein produced by the liver. It binds glucocorticoids and progesterone preferentially and determines the amounts of these steroids that are non-protein bound and accessible to target cells (Lewis *et al*. 2005, Lin *et al*. 2010, Perogamvros *et al*. 2012, Bolton *et al*. 2014, Lei *et al*. 2015). In mammals, CBG is decorated by as many as six *N*-glycans that account for about 30% of its overall mass (Sumer-Bayraktar *et al*. 2011), and at least four of the consensus sites for *N*-glycosylation are in conserved positions.

Glycosylation of secreted proteins like CBG is achieved by the sequential actions of glycosidases and glycosyltransferases within the endoplasmic reticulum and Golgi and may influence protein folding and conformation through interactions between glycan moieties and specific amino acid residues (Mitra *et al*. 2006, Aebi 2013, Hebert *et al*. 2014). Addition of *N*-glycans also affects post-translational quality-control mechanisms; intracellular trafficking that can influence secretion; protein stability, solubility and plasma half-life, as well as interactions with plasma membrane receptors, carbohydrate-binding proteins (lectins) and proteases (Mitra *et al*. 2006, Aebi 2013, Hebert *et al*. 2014).

In human CBG, site-specific utilization and processing of *N*-linked oligosaccharide chains influences its secretion, and glycosylation of N238 influences its steroid-binding activity (Avvakumov *et al*. 1993, Avvakumov & Hammond 1994*a*). Others have confirmed that *N*-glycosylation of CBG is essential for its high-affinity steroid-binding activity through comparisons of CBG expressed using *E. coli* vs the glycosylated protein isolated from serum (Chan *et al*. 2013), and this highlights the importance of using glycosylated CBG for studies of its functional properties. Analysis of the oligosaccharides attached to each of the *N*-glycosylation sites of human CBG has confirmed their differential utilization, as well as variation in the types of oligosaccharides attached to them (Avvakumov & Hammond 1994*a*, Sumer-Bayraktar *et al*. 2011), both of which contribute to the heterogeneity in its apparent molecular size (Sumer-Bayraktar *et al*. 2011). Similar studies have not been performed with rat CBG, but it appears to be more extensively sialylated than human CBG (Blithe *et al*. 1992). Pregnancy-specific glycoforms of human CBG with a higher degree of sialylation, branching and occupancy (Mitchell *et al*. 2004), and a higher affinity for syncytiotrophoblasts cell membranes (Strel’chyonok & Avvakumov 1991), suggest roles for CBG during pregnancy. In addition, treatment with dexamethasone alters the glycosylation profile of CBG in fetal sheep (Berdusco *et al*. 1993), and dexamethasone, thyroxin, insulin and estradiol have all been reported to alter the types and levels of CBG glycoforms secreted by human liver cells (Mihrshahi *et al*. 2006).

Unlike most other structurally related serine proteinase inhibitor (SERPIN) clade A family members, human CBG (SERPINA6) does not inhibit proteases (Law *et al*. 2006). However, as for other SERPINs, the human (Gardill *et al*. 2012) and rat (Klieber *et al*. 2007) CBG structures comprise a reactive center loop (RCL) that is cleaved by proteases. Proteolysis of the RCL of human CBG by neutrophil elastase causes a conformational change that markedly decreases its steroid-binding affinity and is thought to promote the delivery of cortisol to sites of inflammation (Hammond *et al*. 1990, Lin *et al*. 2009). A metalloprotease (LasB), secreted by the pathogen *Pseudomonas aeruginosa,* may also contribute to the localized release of cortisol from CBG at sites of infection through RCL cleavage (Simard *et al*. 2014). Although chymotrypsin cleaves the RCL of human CBG, the physiological relevance of this is unclear (Lewis & Elder 2014). The RCL of CBG in some species, including humans and rats, contains an *N*-glycosylation site, the relative position of which varies between species (Lin *et al*. 2010). The *N*-glycosylation site in the RCL of human CBG has been estimated to be ~85% utilized (Sumer-Bayraktar *et al*. 2011), and carbohydrate chains in this position are known to modulate how proteases access and cleave the RCL (Sumer-Bayraktar *et al*. 2016).

We examined how the *N*-glycosylation of CBG influences its production, steroid-binding properties and its sensitivity to proteases. To do so, we altered the glycosylation profiles of human and rat CBGs through mutagenesis of *N*-glycosylation sites; produced CBG mutants in cell lines with deficiencies in their glycosylation machinery; blocked *N*-glycosylation during synthesis and enzymatically removed *N*-linked oligosaccharides from secreted CBGs.

## Materials and methods

### Production of glycosylation-deficient CBGs

**Figure 1:**
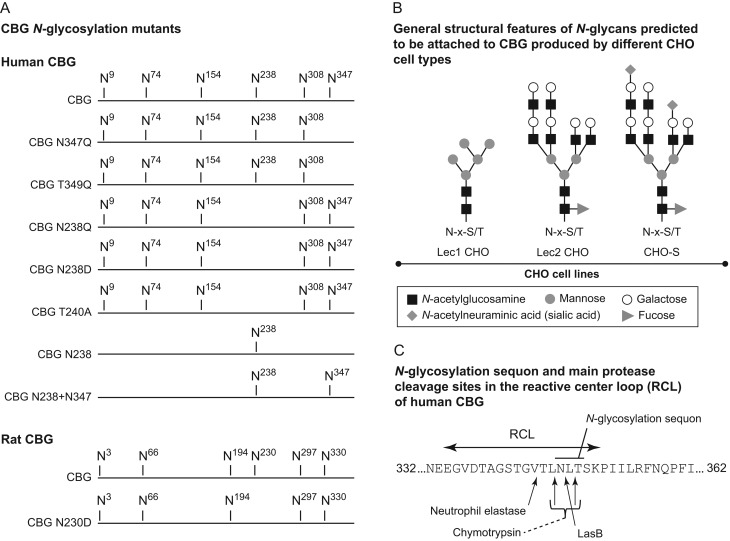
Positions and types of *N*-glycans attached to the human and rat CBGs analyzed, and the RCL sequence of human CBG. (A) Human and rat CBGs and the glycosylation-deficient mutants studied. Human CBG mutants include: CBG N347Q and a naturally occurring variant CBG T349A (Simard *et al*. 2015) in which the *N*-glycosylation site within the RCL is disrupted; CBG N238Q, CBG N238D and CBG T240A in which the *N*-glycosylation site at N238 was disrupted; CBG N238 containing only one *N*-glycosylation site and CBG N238+N347 containing only two carbohydrate chains. Rat CBG N230D that disrupts the* N*-glycosylation site at N230. (B) General structural features of *N*-glycans predicted to be attached to human or rat CBG expressed by glycosylation-competent CHO-S cells or glycosylation-deficient CHO cells, i.e. Lec1 cells that lack *N*-acetylglucosaminyltransferase-I and do not synthesize complex or hybrid *N*-glycans or Lec2 cells that lack the CMP-sialic acid Golgi transporter and do not add sialic acid residues. These proposed structures are based on studies of glycoproteins produced by these cell types (Patnaik & Stanley 2006), and some heterogeneity is to be expected. N-x-S/T is the consensus* N*-glycosylation sequon. (C) Sequence of the human CBG RCL showing the locations of cleavage sites for neutrophil elastase, chymotrypsin and the *P. aeruginosa* protease LasB.

Different CBG glycoforms were produced to study how *N*-glycosylation influences the steroid-binding activity of CBG and its sensitivity to proteases (Fig. 1A). The human CBG mutants N347Q, T349A, N238Q, T240A and N238 were prepared as described (Avvakumov *et al*. 1993, Simard *et al*. 2015). Human and rat CBG cDNAs within pRc/CMV or pcDNA3 expression vectors (Invitrogen), respectively, were also subjected to site-directed mutagenesis to disrupt specific consensus *N*-glycosylation sites using a QuikChange II Site-Directed Mutagenesis Kit (Agilent) and complementary pairs of mutagenic oligonucleotide primers (mutated nucleotide(s) in lower case), as indicated: human CBG N238D (5′-GATGAACTACGTGGGCgATGGGACTGTCTTCTTC); human CBG N238+N347 produced by mutating the human CBG N238 expression plasmid (5′-CTCCACTGGGGTCACCCTAaAcCTGACGTCCAAGCCTATCATC) and rat CBG N230D (5′-AGATGGACTATGTGGGAgATGGAACTGCCTTCTTCATTC). The mutated cDNAs were sequenced to ensure only the targeted mutations had occurred and were expressed after stable transfection of Chinese Hamster Ovary (CHO-S) cells (Gibco #11619-012) or CHO cell lines, Lec1 (ATCC CRL-1735) and Lec2 (ATCC CRL-1736), with defined defects (Patnaik & Stanley 2006, North *et al*. 2010) in glycosylation (Fig. 1B). Transfection and cell culture conditions for stable expression, as well as the semi-purification of secreted CBGs by fast protein liquid chromatography (FPLC) using a HiTrap QFF column, were as described previously (Simard *et al*. 2015).

In addition, CHO cells producing recombinant CBGs were cultured in the presence of tunicamycin (Calbiochem) to generate unglycosylated CBG (Fig. 1B). To accomplish this, 5 µg/mL of tunicamycin was added to culture media for 96 h. Culture medium containing CBG was then harvested, centrifuged to remove debris, filtered using 0.22 µm filters, concentrated ~4-fold using Amicon Ultra 3K centrifugal filters (Millipore) and buffer exchanged with 20 mM Tris (pH 8). PNGase F and Endo H (both from New England Biolabs) were used to remove *N*-linked oligosaccharides (Fig. 1B) to test the effect of glycan removal on CBG steroid-binding activity. To deglycosylate human CBG with PNGase F, 1 µL (500 units) of enzyme was added to 100 µL of ~3–5 nM purified CBG in 20 mM Tris (pH 8) and incubated for 16 h at 37°C. To deglycosylate CBG with Endo H, 1 µL (500 units) of enzyme was added to 500 µL of ~30 nM CBG in concentrated and buffer-exchanged Lec1 CHO cell medium and incubated for 16 h at 37°C.

### Steroid-binding activity measurements

A radiolabeled-steroid saturation assay was used to detect and measure CBG in concentrated and buffer-exchanged culture media or after chromatographic purification (Simard *et al*. 2015). In brief, steroid-binding capacity measurements and Scatchard analyses of steroid-binding affinity were performed using [^3^H]cortisol or [^3^H]corticosterone (PerkinElmer Health Sciences) as the labeled ligands for human and rat CBGs, respectively, and dextran-coated charcoal to separate bound from free [^3^H]-labeled steroids (Hammond & Lahteenmaki 1983).

### Western blot analysis

Western blots were performed to assess the amounts or integrity of human and rat CBGs after tunicamycin treatment, deglycosylation by PNGase F or Endo H or incubations with proteases. Samples were subjected to sodium dodecyl sulfate-polyacrylamide gel electrophoresis (SDS-PAGE) and transferred to polyvinylidene fluoride membranes. Western blots were incubated with 1:5,000 dilutions of rabbit anti-human CBG antiserum (Robinson *et al*. 1985) or a rabbit anti-mouse CBG antiserum that recognizes rat CBG (Hill *et al*. 2016), followed by a 1:10,000 horseradish peroxidase-labeled goat antirabbit IgG antibody (Sigma-Aldrich). Immunoreactive CBG was detected using ECL Prime Western Blotting Detection Reagent and an ImageQuant LAS4000 (GE Healthcare).

### Proteolysis of the CBG RCL

The CBG glycoforms were tested for their sensitivity to proteolysis after incubation with proteases (neutrophil elastase, bovine α-chymotrypsin, LasB) that specifically target the CBG RCL (Fig. 1C). The amounts of enzymes used were adjusted to produce ~35–55% reductions in the steroid-binding capacity of the recombinant un-mutated CBGs. Neutrophil elastase (Elastin Products) was reconstituted at 0.1 µg/µL in a buffer containing 0.05 M NaAc (pH 5) and 0.1 M NaCl. Indicated amounts were added to CBG samples in 100 µL 20 mM Tris (pH 8) and incubated for 10 min at 37°C followed by the addition of 5 mM phenylmethanesulfonyl fluoride to stop reactions, prior to steroid-binding capacity assays or SDS-PAGE. Bovine α-chymotrypsin (type II from pancreas; Sigma-Aldrich) was reconstituted at 1 µg/µL in 0.1 M Tris–HCl (pH 7.5), 0.5 M NaCl. Indicated amounts were incubated with CBG samples as described for neutrophil elastase, prior to steroid-binding capacity assays or SDS-PAGE. Medium from a culture of *Pseudomonas aeruginosa* was used as a source of LasB (Simard *et al*. 2014). Indicated amounts were added to CBG samples in 100 µL 20 mM Tris (pH 8) and incubated (3 h at 37°C) followed by addition of 5 mM EDTA to stop reactions, prior to steroid-binding capacity assays or SDS-PAGE.

### Statistical analysis

One-way ANOVA followed by Tukey’s or Dunnett’s multiple comparisons tests or two-way ANOVA followed by Bonferroni tests were performed as indicated using GraphPad Prism 5 software (GraphPad Software). A *P* value <0.05 was considered significant.

## Results

### Implications of *N*-glycosylation on CBG steroid-binding activity

**Figure 2:**
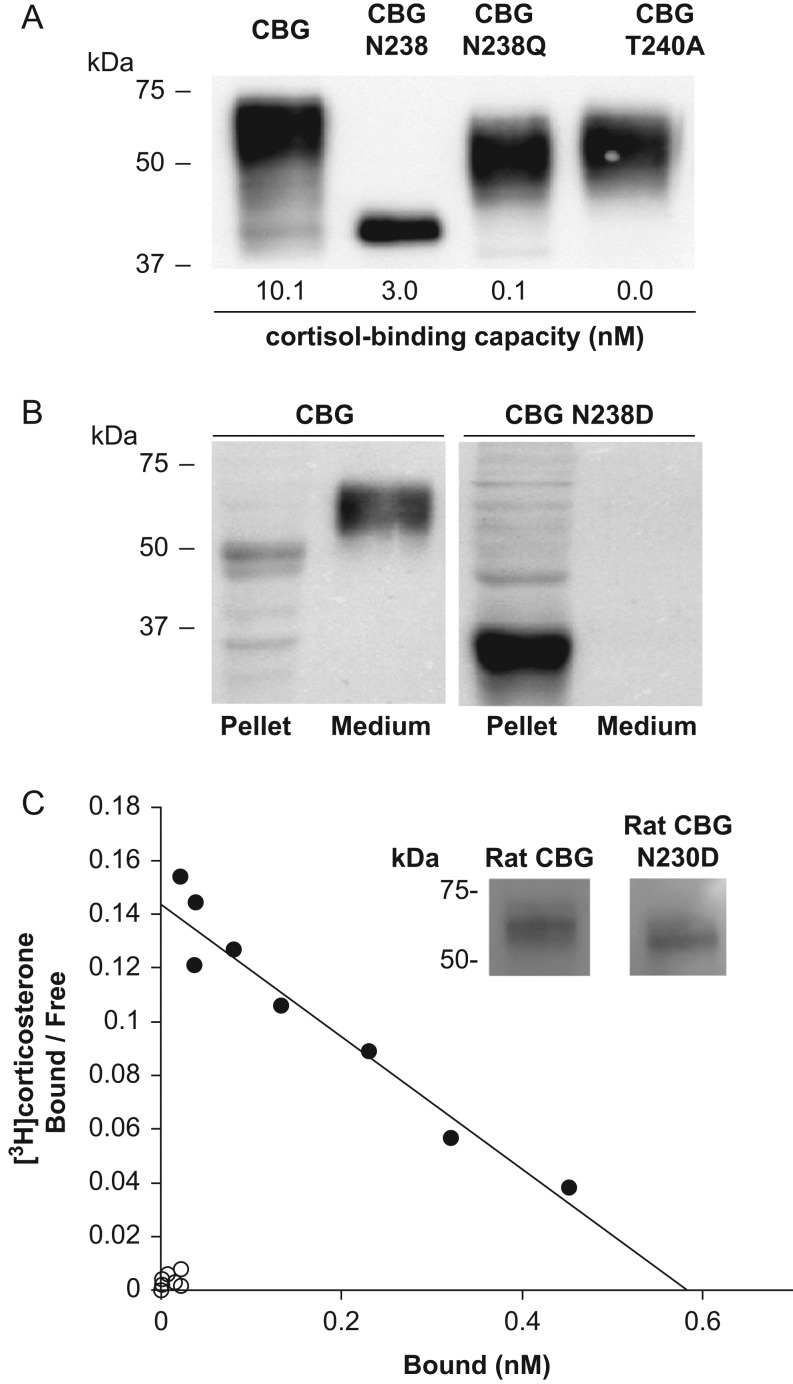
*N*-Glycosylation at N238 in human CBG and N230 in rat CBG is required for high-affinity steroid binding. (A) Human CBG N238Q and CBG T240A produced by CHO-S cells and FPLC-purified show the expected reduction in molecular size when compared to fully glycosylated CBG. These mutants have much lower cortisol-binding capacities as compared to CBG or CBG N238 with only a single *N*-glycan. (B) Human CBG N238D was undetectable in the culture media but present in cell pellet extracts of transfected CHO-S cells. (C) Loss of *N*-glycosylation at N230 in rat CBG N230D reduces its molecular size and disrupts its steroid binding. Western blot of rat CBG and rat CBG N230D in 5 µL of concentrated and buffer-exchanged CHO-S cell culture media demonstrates that similar amounts of both proteins were secreted. Scatchard analysis of similar amounts of rat CBG (black circles) and rat CBG N230D (white circles) adjusted based on their immunoreactivity. Positions of molecular size markers (kDa) are shown.

When the relative positions of *N*-glycosylation sites in human and rat CBGs are compared, only one of the sites is not in a conserved position; N154 in human CBG and N194 in rat CBG (Fig. 1A). As noted previously (Avvakumov *et al*. 1993), disruption of the *N*-glycosylation site of human CBG at N238 by substitution of Asn 238 with Gln or Thr 240 with Ala causes a major loss of cortisol-binding capacity when expressed in CHO cells, whereas a human CBG mutant with only a single *N*-glycosylation site at N238 (CBG N238) bound steroid appropriately in relation to its immunoreactivity on a Western blot (Fig. 2A). We also substituted N238 in human CBG with Asp, but the mutated protein was not secreted into the culture medium by CHO cells and accumulated within the cells, presumably as a misfolded and partially degraded protein. We base this on the observation that the CBG N238D that accumulates in the CHO cell pellets runs close to the 37 kDa size marker, whereas we would have expected it to run in excess of 42 kDa (the molecular size of the CBG polypeptide) because its five other glycosylation sites are intact (Fig. 2B). However, when the corresponding *N*-glycosylation site at N230 in rat CBG was disrupted in this way, the rat CBG N230D was secreted but had barely detectable steroid-binding activity (Fig. 2C).

**Figure 3:**
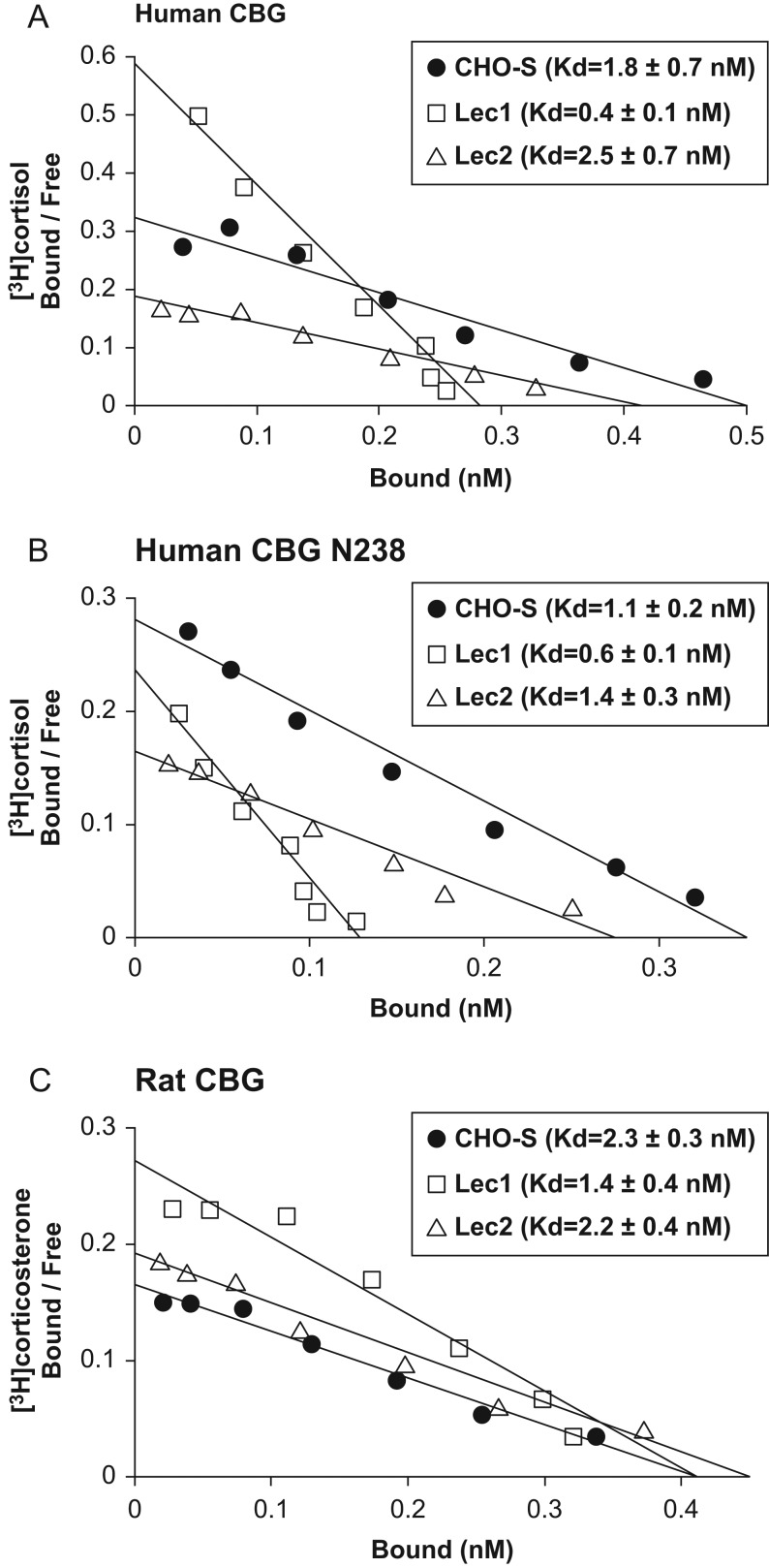
Differences in *N*-glycosylation influence the steroid-binding affinity of human and rat CBGs. Scatchard analyses of [^3^H]cortisol binding to (A) human CBG and (B) a human CBG mutant with only a single *N*-linked glycan at N238, and (C) [^3^H]corticosterone binding to rat CBG. Human and rat CBGs were produced by CHO-S cells, as well as by glycosylation-deficient Lec1 or Lec2 cells, and purified by FPLC for analysis, as described (Simard *et al*. 2015). Representative data and linear fit are shown and mean ± s.d. dissociation constants (Kd) are shown in parentheses for replicate experiments (*n* = 5). One-way ANOVA followed by Tukey’s multiple comparisons test for each group revealed that the steroid-binding affinity of the CBGs produced in Lec1 cells is significantly higher than those produced in CHO-S or Lec2 cells (A and B, *P* < 0.01; C, *P* < 0.05).

The steroid-binding properties of human and rat CBGs produced by CHO cell lines (Fig. 1B) that are glycosylation competent (CHO-S cells), deficient in *N*-acetylglucosaminyltransferase (Lec1 cells), or lack the CMP-sialic acid Golgi transporter required for sialylation (Lec2 cells), were determined by Scatchard analysis after FPLC chromatographic purification (Fig. 3). When produced in Lec1 cells, human CBG (Fig. 3A) and human CBG N238 (Fig. 3B) showed ~4-fold (*P* < 0.05) and ~2-fold (*P* < 0.05) higher binding affinities for cortisol, respectively, when compared to their counterparts produced in CHO-S or Lec2 cells. These data indicate that the composition of an *N*-glycan at N238 specifically influences the steroid-binding activity of human CBG. Moreover, when rat CBG was examined in this way, a similar ~2-fold (*P* < 0.05) increase in its affinity for corticosterone was observed when expressed in Lec1 cells vs CHO-S or Lec2 cells (Fig. 3C).

**Figure 4:**
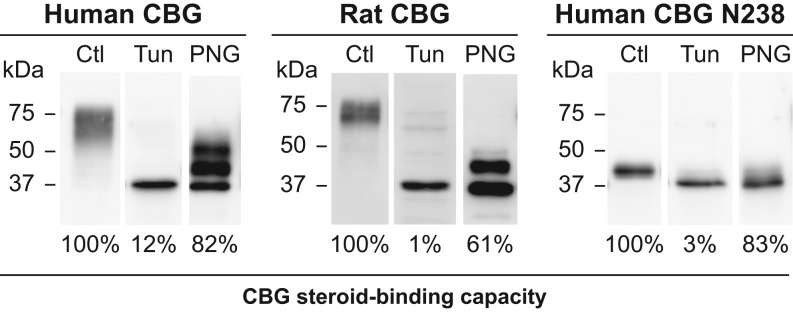
Effects of inhibiting glycosylation or removing *N*-glycans on human and rat CBG steroid binding. The CBGs were expressed in CHO-S cells in the presence or absence of the *N*-glycosylation inhibitor tunicamycin (Tun) and the culture media were concentrated and buffer-exchanged for Western blotting and steroid-binding capacity measurements. Reductions in apparent molecular size and loss of micro-heterogeneity are consistent with the absence of glycosylation. The steroid-binding capacities of human and rat CBGs produced by tunicamycin-treated CHO-S cells were compared as a percentage (%) of those produced by untreated (Ctl) CHO-S cells after adjusting their amounts based on Western blotting. Similar amounts of human CBG, rat CBG, and human CBG N238 produced in untreated CHO-S cells were also incubated with PNGase F (PNG) to remove *N*-glycans. The amounts of PNGase F and incubation time were optimized to ensure that removal of *N*-glycans was as complete as possible, and similar results were observed when 500 units of PNGase F treatment were used for 3 h or 16 h at 37°C. Western blotting was used to assess the efficacy of deglycosylation and steroid-binding capacities were expressed as a percentage (%) of those obtained for the untreated (Ctl) samples. Positions of molecular size markers (kDa) are indicated.

To determine how the inhibition of glycosylation influences the steroid-binding activity of rat and human CBGs, they were produced in CHO-S cells in the presence of tunicamycin. This treatment reduced CBG production in all cases to 10–20% of that produced by untreated cells, as assessed by Western blotting, and it clearly blocked the glycosylation of CBG (Fig. 4). However, in relation to their immunoreactivity by Western blotting, the steroid-binding capacity of unglycosylated human CBG, rat CBG and human CBG N238 was reduced by 88–99%, as compared to their glycosylated counterparts (Fig. 4).

Human and rat CBGs were incompletely deglycosylated after PNGase F treatment suggesting that *N*-linked glycans in specific locations resisted excision (Fig. 4). Reductions in steroid-binding capacity were observed for human (18%) and rat (39%) CBGs after deglycosylation with PNGase F (Fig. 4), but their steroid-binding affinities (human CBG: Kd control = 1.1 nM, Kd deglycosylated = 1.3 nM; rat CBG: Kd control = 2.1 nM, Kd deglycosylated = 1.8 nM) determined by Scatchard analysis were not altered. Importantly, however, PNGase F effectively removed the single *N*-linked oligosaccharide from human CBG N238 (Fig. 4), and the single *N*-glycan at this location is therefore fully accessible to the enzyme. Moreover, while PNGase F removal of the *N*-linked glycan from CBG N238 caused a small loss (17%) in steroid-binding capacity, its high steroid-binding affinity (Kd control = 0.7 nM, Kd deglycosylated = 1.1 nM) was retained (Fig. 4).

**Figure 5:**
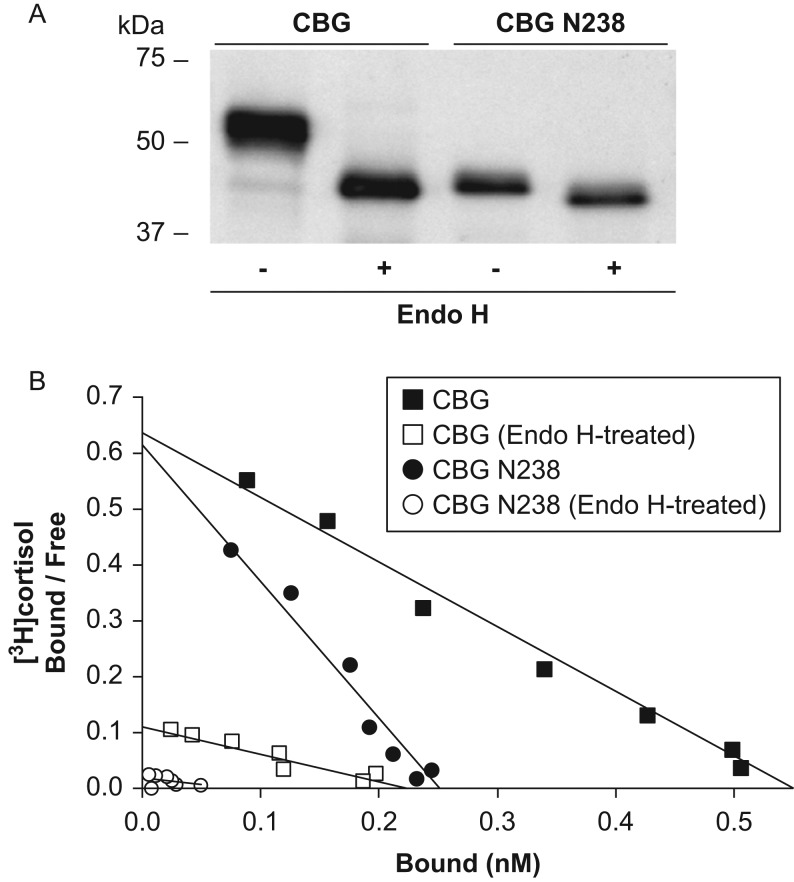
Deglycosylation of human CBG with Endo H reduces its steroid-binding affinity. (A) Western blot showing that fully glycosylated CBG and CBG N238 in concentrated and buffer-exchanged culture media from Lec1 CHO cells are completely deglycosylated after treatment with Endo H. Positions of molecular size markers (kDa) are indicated. (B) Representative Scatchard analyses showing that deglycosylation with Endo H leads to major decreases in cortisol-binding affinity (Kd), which were determined in two separate experiments for CBG (untreated, 0.8 nM and 0.5 nM vs Endo H-treated, 1.9 nM and 1.9 nM) and CBG N238 (untreated, 0.4 nM and 1.0 nM vs Endo H-treated, 4.2 nM and 4.8 nM).

Given that PNGase F deglycosylation deamidates asparagine residues converting them into aspartic acid, we used Endo H to deglycosylate human CBG because it leaves asparagine residues intact with only a single *N*-acetylglucosamine attached to them (Fig. 5A). However, as observed for other glycoproteins (Freeze & Kranz 2010), CBGs expressed in CHO-S or Lec2 CHO cells contain complex or hybrid *N*-glycans that are resistant to Endo H cleavage, and we therefore used Lec1 CHO cells for this purpose. Unlike PNGase F, the Endo H-mediated deglycosylation of human CBG or human CBG N238 produced by Lec1 cells results in ~2–10 fold losses in steroid-binding affinity, respectively (Fig. 5B).

### Proteolysis of CBG glycoforms

**Figure 6:**
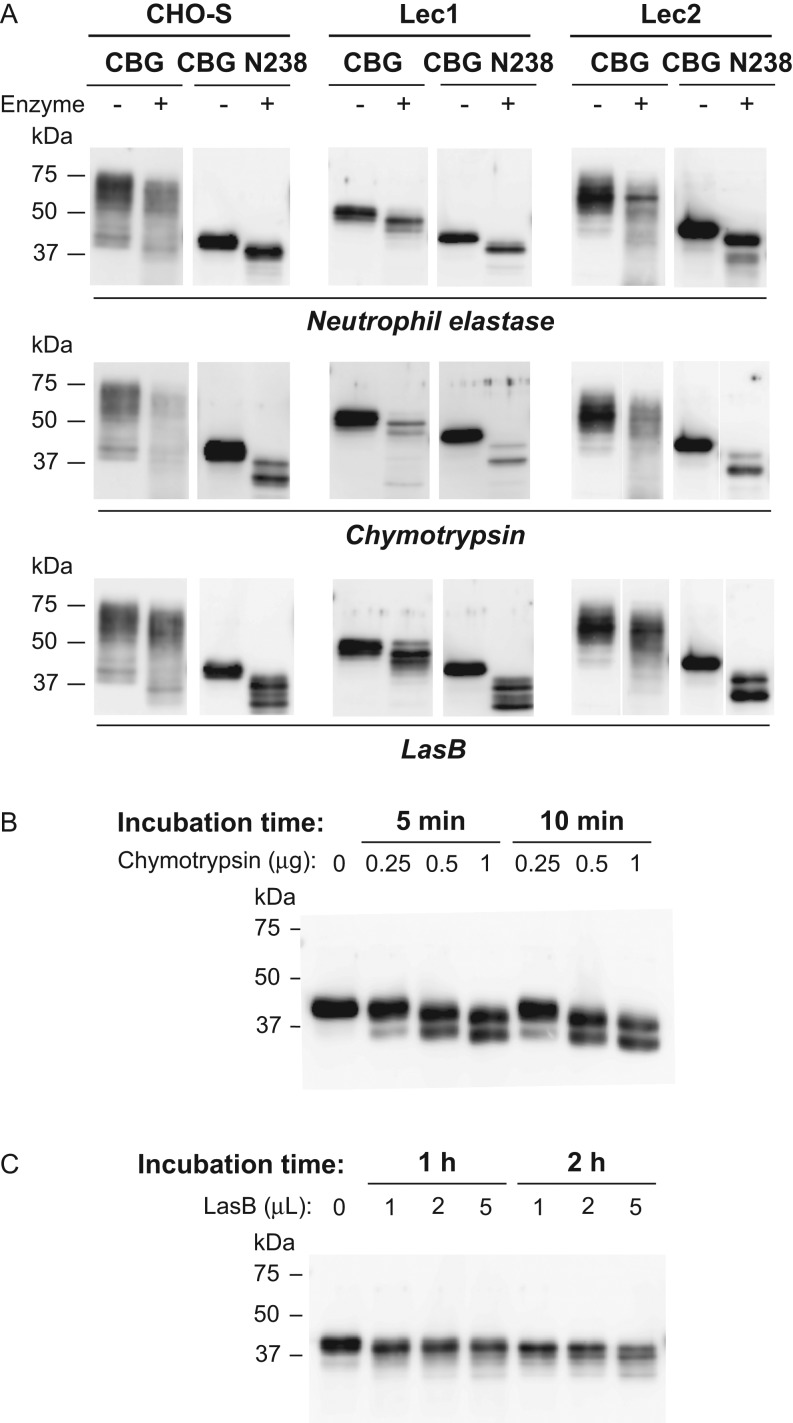
Impact of quantitative and qualitative differences in *N*-glycosylation on proteolysis of human CBG. (A) FPLC-Purified human CBG and CBG N238 produced in CHO-S, Lec1, or Lec2 cells were incubated with neutrophil elastase (0.1 µg for 10 min at 37°C), chymotrypsin (1 µg for 10 min at 37°C), or *P. aeruginosa* media (5 µL for 3 h at 37°C) containing LasB and subjected to Western blotting. Reductions (~5–10 kDa) in molecular size were observed after incubation with proteases. (B and C) Western blots of human CBG N238 after limited incubation times with different amounts of chymotrypsin or LasB. Positions of molecular size markers (kDa) are indicated.

Human neutrophil elastase (Lin *et al*. 2009), bovine chymotrypsin (Lewis & Elder 2014) and the bacterial protease LasB (Simard *et al*. 2014) preferentially cleave the RCL of human CBG in specific locations (Fig. 1C). When examined by Western blotting, fully glycosylated CBG, and non-sialylated CBG expressed in Lec2 cells, display considerable size heterogeneity, but after incubation with neutrophil elastase there is a general reduction in their molecular size by ~5 kDa, which is consistent with proteolysis of the RCL (Fig. 6A). The CBG produced in Lec1 cells exhibits far less size heterogeneity, and an ~5 kDa reduction in molecular size was evident after incubation with neutrophil elastase (Fig. 6A). This was also observed with human CBG N238 with only a single *N*-linked oligosaccharide (Fig. 6A).

When these human CBG glycoforms were incubated with chymotrypsin or LasB, the protease activities appeared to be less specific than observed for neutrophil elastase (Fig. 6A), with evidence of additional sites of proteolysis (Fig. 6A). While this was not readily seen with fully glycosylated CBG due to its size heterogeneity, a second major proteolytic fragment was observed when the CBG N238 mutant or the CBG glycoforms produced by Lec1 cells were tested (Fig. 6A). However, the appearance of these additional proteolytic products depended on the amounts of chymotrypsin or LasB used and the incubation times (Fig. 6B and C). When these latter variables were examined, an ~5 kDa reduction in molecular size occurred initially, consistent with RCL cleavage, followed by additional proteolysis and a further size reduction of ~5–10 kDa (Fig. 6B and C).

### Steroid-binding activities of CBG glycoforms after RCL proteolysis

**Figure 7:**
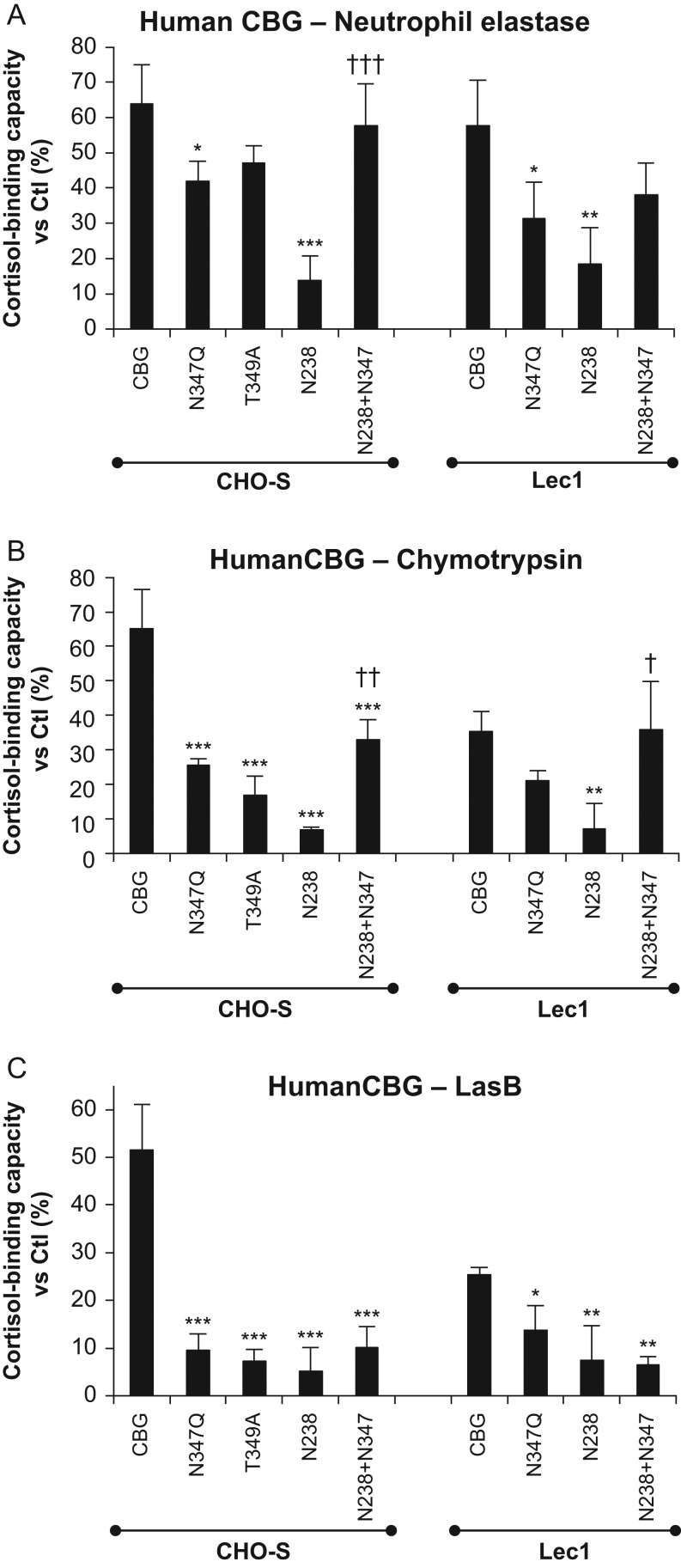
Impact of *N*-glycosylation on human CBG steroid binding after incubations with proteases that target the RCL. FPLC-Purified human CBG and CBG glycosylation mutants produced in CHO-S and Lec1 CHO cells were incubated with (A) neutrophil elastase (25 ng for 10 min at 37°C), (B) chymotrypsin (0.25 µg for 10 min at 37°C), or (C) *P. aeruginosa* media (2 µL for 3 h at 37°C) containing LasB. The amounts of enzymes and incubation times were adjusted to obtain ~35–50% of residual steroid-binding activity for un-mutated CBG, and similar amounts of un-mutated and mutated CBGs were tested in relation to their steroid-binding capacity values. Cortisol-binding capacity values are expressed as a percentage (%) of values for the respective untreated samples (Ctl). One-way ANOVA followed by Dunnett’s multiple comparisons test (for CHO and Lec1 groups separately, in A, B, and C) were performed to assess differences (*P < 0.05; **P < 0.01; ***P < 0.001) between the un-mutated CBG and the CBG glycosylation mutants. One-way ANOVA followed by Tukey’s multiple comparisons test were performed to assess differences (^†^*P* < 0.05; ^††^*P* < 0.01; ^†††^*P* < 0.001) between CBG N238 and CBG N238+N347 in each group.

**Figure 8:**
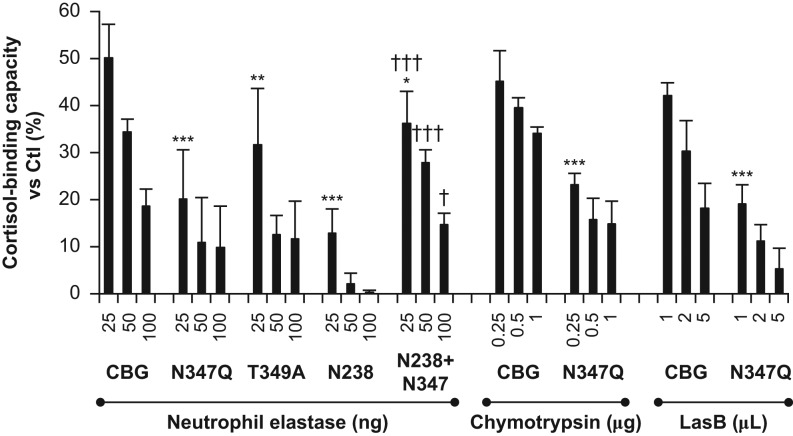
Evidence that *N*-glycosylation at specific sites protects against CBG proteolysis and loss of steroid-binding activity. FPLC-Purified human CBG and several CBG glycosylation-deficient mutants were incubated with neutrophil elastase (25, 50 and 100 ng for 10 min at 37°C), chymotrypsin (0.25, 0.5 and 1.0 µg for 10 min at 37°C) or LasB (1, 2 and 5 µL for 3 h at 37°C). Cortisol-binding capacity values are expressed as a percentage (%) of values obtained for the respective untreated samples (Ctl). Two-way ANOVA followed by Bonferroni tests were performed for each group (neutrophil elastase, chymotrypsin, and LasB) to evaluate the effects of enzyme concentration on loss of cortisol-binding capacity, and differences between CBG and the various CBG glycosylation-deficient mutants. When compared with the un-mutated CBG, all of the CBG glycosylation mutants tested had significantly greater reductions in cortisol-binding capacity at the lowest concentrations of enzymes tested (*P < 0.05; **P < 0.01; ***P < 0.001), and further reductions were observed at higher enzyme concentrations in all cases. The greatest losses in cortisol-binding capacity were observed after treatment of the CBG N238 mutant with only one *N*-glycosylation site, and this was significantly abrogated (^†^*P* < 0.05; ^†††^*P* < 0.001) by the presence of an *N*-glycosylation site within the RCL at N347 as well as at N238.

Cleavage of the human CBG RCL by neutrophil elastase, chymotrypsin or LasB leads to a loss of high-affinity steroid binding (Hammond *et al*. 1990, Lewis & Elder 2014, Simard *et al*. 2014), and we examined how the *N*-glycosylation of CBG influences the ability of these proteases to act in this way. Because the targeted RCL cleavage of CBG by these proteases is exceptionally efficient (Hammond *et al*. 1990, Lewis & Elder 2014, Simard *et al*. 2014), we titrated the amounts of CBG and proteases used in the incubations to achieve a limited cleavage of the un-mutated human and rat CBGs, as evidenced by ~35–55% reductions in their steroid-binding activity. In these experiments, most human CBG glycosylation-deficient mutants showed a greater decrease in steroid binding than the un-mutated CBG after incubation with the proteases, the greatest decreases being observed with LasB (Fig. 7A, B and C). Notably, human CBG N238 showed the greatest losses of cortisol binding after incubation with the enzymes tested. However, addition of an *N*-glycosylation site at N347 within the RCL of CBG N238 abrogated the losses of cortisol-binding activity, especially after incubation with neutrophil elastase or chymotrypsin (Fig. 7A and B). Similar effects were observed for the CBG mutants produced in CHO-S or Lec1 cells (Fig. 7). The protective effect of an *N*-glycosylation site at N347 was not significant in the experiments where LasB was tested (Fig. 7C). This likely reflected the much greater overall losses of cortisol-binding activity observed, and a follow-up experiment was conducted using a range of enzyme concentrations with native CBG and the CBG mutants expressed in CHO-S cells (Fig. 8).

By incubating the CBG glycosylation mutants with different amounts of neutrophil elastase the presence of an *N*-glycosylation site at N347 again consistently protected against loss of cortisol-binding capacity (Fig. 8). Moreover, the same general profile of cortisol-binding capacity losses observed in the previous experiment (Fig. 7) occurred after incubation with different amounts of neutrophil elastase: CBG N238 > CBG N347Q ≈ CBG T349A > CBG N238+N347 > CBG. The RCL mutant CBG N347Q also showed significant losses of steroid binding when compared to un-mutated CBG after incubation with different amounts of chymotrypsin or LasB (Fig. 8). Together, these data suggest a protective effect of the RCL carbohydrate, as well as a more global protective effect of other *N*-glycans on the sensitivity of CBG to proteolysis especially by neutrophil elastase and chymotrypsin.

## Discussion

The contribution that *N*-glycans make to the structure and function of plasma glycoproteins like CBG is not well appreciated. The crystal structures of *E. coli*-expressed human and rat CBGs correspond well to other SERPINA structures (Klieber *et al*. 2007, Gardill *et al*. 2012), but the steroid-binding affinities of CBGs produced in *E. coli* are ~10-fold lower than those of the natural proteins that are extensively *N*-glycosylated (Lin *et al*. 2010, Vashchenko *et al*. 2016). Production of CBG in transformed human cell lines, like HepG2 cells, or in other mammalian cells does not perfectly mimic the types of *N*-glycan additions that occur in normal liver cells (Butler & Spearman 2014), but CHO cells have been used extensively for this purpose. Moreover, CHO cell lines with deficiencies in specific enzymatic steps in *N*-glycosylation pathways (Patnaik & Stanley 2006) allow studies of how quantitative and qualitative differences in *N*-glycosylation affect the functional properties of glycoproteins like CBG.

**Figure 9:**
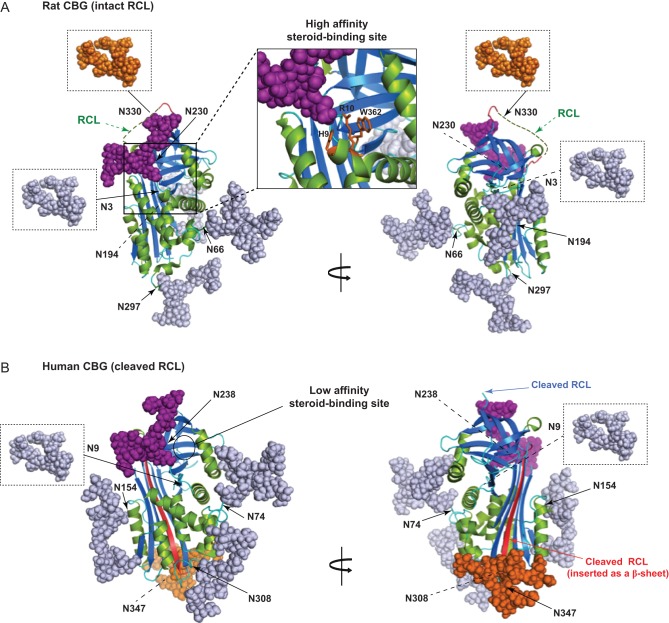
Structural models showing the extent of *N*-glycosylation of rat and human CBGs in stressed (high affinity with intact RCL) and relaxed (low affinity with cleaved RCL) conformational states. (A) The rat CBG in its stressed conformation (PDB ID 2V95), with a close-up of the steroid-binding site with the positions of H9, R10, and W362 colored *orange*. (B) Human CBG in its relaxed conformation (PDB ID 4BB2), with its cleaved RCL inserted as a β-sheet colored *red*. Models were generated using the PyMOL Molecular Graphics System software (http://pymol.org) and the online tool for *in silico* glycosylation of proteins GlyProt (http://glycosciences.de). The* N*-glycans attached in various positions are shown based on their reported most frequent compositions in human CBG (Sumer-Bayraktar *et al*. 2011). *N*-glycans that could not be added because they are in regions of disorder in the crystal structures are indicated in boxes (at N3 and N330 in rat CBG, and at N9 in human CBG). Notably, the comparison between stressed and relaxed CBG structures shows the displacement of N347 with its associated* N*-glycan in human CBG after RCL cleavage. Both structures show β-sheets (*blue*), α-helixes (*green*), loops (*cyan*), and *N*-glycans in *gray* except those at N238 in human CBG and N230 in rat CBG (*purple*), and those attached to the cleaved RCL of human CBG (at N347) and the intact RCL of rat CBG (at N330), which are colored *orange*.

To illustrate how *N*-glycans contribute to the overall physical properties of CBG, we applied *in silico* glycan structure modeling to the crystal structures of rat (Klieber *et al*. 2007) and human (Gardill *et al*. 2012) CBGs in their ‘stressed’ (RCL intact) and ‘relaxed’ (RCL cleaved) SERPIN conformations, respectively (Fig. 9). This shows the extent to which *N*-glycans decorate the surface of the proteins, and how amino acids and oligosaccharide chains might interact to induce conformational changes with functional consequences. This extensive degree of *N*-glycan decoration is not generally appreciated, but may influence the recognition of surface epitopes by antibodies, and especially monoclonal antibodies raised using synthetic peptide antigens. The ‘protective shield’ that the *N*-glycans provide may also restrict interactions with proteases to functionally relevant sites within the RCL.

Human CBG produced in insect Sf9 cells that produce only oligosaccharides of the high-mannose type bound cortisol with high affinity, while unglycosylated CBG produced in Sf9 cells in the presence of tunicamycin was inactive (Ghose-Dastidar *et al*. 1991). Our finding that unglycosylated human and rat CBGs produced in tunicamycin-treated CHO cells have virtually undetectable steroid-binding activity confirms the latter effect, and supports the assumption that *N*-glycosylation is required for the structural acquisition of a high affinity steroid-binding site during synthesis. Additional evidence for this was obtained through disruption of the sequon responsible for *N*-glycosylation at N238 in human CBG or N230 in rat CBG through mutagenesis in various ways, because the resulting mutants were characterized by very low steroid-binding affinities when expressed in CHO cells.

Removal of the single *N*-glycan from human CBG N238 with PNGase F did not adversely alter its steroid-binding affinity, implying that this carbohydrate chain can be removed without perturbation of the binding site, as noted previously (Avvakumov & Hammond 1994*a*). However, it appears that trimming of the *N*-glycan at N238 in human CBG to its initial *N*-acetylglucosamine with Endo H causes a 10 fold loss in steroid binding. This difference in the effects of these two endoglycosidases is interesting because PNGase F removes all sugar residues and deamidates the Asn converting it to an Asp residue, while Endo H leaves the Asn intact with a single *N*-acetylglucosamine attached to it. It could be argued that the PNGase F-mediated conversion of Asn to Asp somehow prevents perturbation of the steroid-binding site once it is appropriately configured. If this is the case, however, the protein may need to be already appropriately folded with a high affinity-binding site because rat CBG N230D was secreted at a normal level but had no detectable steroid binding: an observation that again suggests that an *N*-glycan at this position is necessary for the acquisition of a high affinity steroid-binding site during synthesis.

We also explored the possibility that qualitative differences in *N*-glycosylation affect the steroid-binding properties of human CBG. Scatchard analyses of CBG glycoforms produced in Lec1 cells suggest that the first two *N*-acetylglucosamine and attached mannose residues, as well as the antennary *N*-acetylglucosamine and attached galactose residues (Fig. 1B), influence steroid-binding affinity. Moreover, because CBG and CBG N238 behaved similarly in this regard, modifications in the glycan chain attached at N238 would appear to be the primary determinant of steroid-binding affinity. The increase in steroid-binding affinity observed for rat CBG produced in Lec1 cells also suggests that carbohydrate composition influences its steroid binding in similar ways. By contrast, the terminal sialic acid residues on the glycan chains do not seem to contribute to this effect, as shown by the absence of significant differences in steroid-binding affinity between samples produced in CHO-S and Lec2 cells. These surprising changes in the steroid-binding affinity of CBG that are linked to qualitative differences in its *N*-glycan additions suggest that its ability to bind steroids varies within the endoplasmic reticulum and Golgi compartments of cells during synthesis. Since steroids are likely present in these subcellular compartments, it is therefore possible that changes in the steroid-binding affinity of CBG during post-translational modifications prior to secretion may also affect folding events and the acquisition of its steroid-binding properties. Conversely, it is possible that many naturally occurring mutations in human CBG, some of which alter its production, steroid-binding properties or sensitivity to proteases (Simard *et al*. 2015), may influence its glycosylation both quantitatively and qualitatively. These observations may also be important given the increasing number and variety of congenital disorders of glycosylation associated with a wide range of clinical phenotypes in humans with specific genetic defects of the glycosylation machinery (Freeze *et al*. 2014).

The amino-terminal *N*-glycosylation sites of human and rat CBGs (N9 in human; N3 in rat) are positioned close to a conserved Arg (R15 in human; R10 in rat) that contributes to the intra-molecular interactions required for the formation of a functional steroid-binding site (Lin *et al*. 2010, Gardill *et al*. 2012). However, site-directed mutagenesis that disrupts *N*-glycosylation at this position has no impact on human CBG production or its steroid-binding properties (Avvakumov *et al*. 1993, Avvakumov & Hammond 1994*a*). Like the *N*-glycan at N238 in human CBG, our experiments demonstrate that *N*-glycosylation at N230 in rat CBG is essential for establishing a high affinity steroid-binding site. Interestingly, an Asn in this location is conserved in the CBGs of all mammalian species and is positioned close to several other highly conserved amino acids that influence steroid binding. For instance, conserved His and Arg residues (H9 and R10 in rat CBG and H14 and R15 in human CBG) are required for high affinity steroid-binding activity (Klieber *et al*. 2007, Simard *et al*. 2015), and both could interact with oligosaccharides attached at N230 in rat CBG or N238 in human CBG (Fig. 9A and B, respectively). Mutation of these two amino-terminal residues causes major losses in steroid binding and it has been proposed that they interact with a critical tryptophan residue in the human (W371) and rat (W362 – see Fig. 9) CBGs that holds steroids within their binding sites (Klieber *et al*. 2007, Lin *et al*. 2010, Simard *et al*. 2015). Thus *N*-glycans at N238 in human CBG or at N230 in rat CBG could influence steroid binding through altering such intra-peptide interactions.

Our data imply that variations in *N*-glycan composition cause changes in protein structure sufficient to alter the steroid-binding pocket, although it has been suggested that limited branching of oligosaccharides at N238, as well as an absence of fucose, may facilitate the access of steroids to their binding site (Sumer-Bayraktar *et al*. 2011). These may be physiologically relevant effects given that CBG glycosylation profiles are altered during pregnancy (Mitchell *et al*. 2004) or after exposure of liver cells to hormones (Mihrshahi *et al*. 2006). Conversely, the composition of *N*-glycans, such as those linked at N238, may also be determined by the surrounding amino acids (Barb *et al*. 2010). For instance, it has been proposed that Trp266 in human CBG influences the processing of the *N*-glycan at N238 and may limit its secretion (Avvakumov & Hammond 1994*b*). Glycan processing of individual sites is also known to be heavily dependent on the secondary and tertiary structures of proteins (Thaysen-Andersen & Packer 2012). It is therefore likely that the composition of *N*-glycans in other locations influences the production and function of CBG, or its recognition by other macromolecules, including antibodies and lectins.

Remarkably, of the six *N*-glycosylation sites in human CBG, N238 appears to be the least (~75% occupied) utilized (Sumer-Bayraktar *et al*. 2011). However, the latter studies were performed using human CBG isolated by an undefined affinity purification method (Affiland SA, Belgium), and this may be relevant in light of our results. For instance, if the CBG had been isolated using a steroid-affinity matrix, only glycoforms with high steroid-binding affinity would be expected in the CBG used for *N*-glycan analysis, and N238 should have been fully occupied by an *N*-glycan, according to our results. Whereas, if the CBG was isolated using an immuno-affinity matrix, this type of discrepancy between our results and the *N*-glycan utilization data (Sumer-Bayraktar *et al*. 2011) implies that CBG glycoforms exist in human blood without an oligosaccharide at N238 and with a very low affinity for steroids.

In its stressed SERPIN conformation, an unstructured exposed RCL is evident in the rat CBG crystal structure (Klieber *et al*. 2007) while the human CBG structure (Gardill *et al*. 2012) shows the relaxed conformational change that occurs when the RCL is cleaved and inserted as a β-sheet (Fig. 9). All the protease cleavage sites within human CBG identified so far are located between positions 344 and 351 in the RCL surrounding the N347 glycosylation site (Fig. 1C). The neutrophil elastase cleavage site is located several residues amino-terminal to N347 (Hammond *et al*. 1990, Pemberton *et al*. 1988), but chymotrypsin (Lewis & Elder 2014) and LasB (Simard *et al*. 2014) both cleave the human CBG RCL at sites flanking N347. While these cleavage sites for neutrophil elastase, chymotrypsin and LasB were identified in fully glycosylated CBG from human serum, the crystal structure of human CBG produced in *E. coli* shows the conformational change that occurs when its RCL undergoes proteolysis and inserts as a β-sheet in the absence of glycosylation (Klieber *et al*. 2007, Gardill *et al*. 2012).

Our model of the RCL-cleaved human CBG structure illustrates the extent of *N*-glycosylation and the locations of *N*-glycans (Fig. 9). The carbohydrate chain at N347 is also shown at the carboxy-terminus of the inserted RCL sequence as a β-sheet. This model is based on the assumption that a carbohydrate chain in this position does not hinder RCL insertion, and it remains to be determined if this is correct. When the RCL of CBG is cleaved by neutrophil elastase this is not an issue because cleavage occurs amino-terminal to N347 and the inserted RCL would not have a sugar chain attached, but insertion of the cleaved RCL may be hindered to some extent by its *N*-glycosylation after cleavage by chymotrypsin or LasB. Nevertheless, when glycosylated human CBG is incubated with chymotrypsin or LasB both proteases cause substantial losses in steroid binding consistent with cleavage and insertion of the RCL. The proximity between protease cleavage sites and the oligosaccharide attached to N347 in human CBG prompted us to examine how *N*-glycans at this and other positions influence the activities of proteases that cleave the human CBG RCL.

When the human CBG N238 glycosylation-deficient mutant was produced in CHO-S or Lec1 cells, it consistently showed the lowest steroid-binding capacity among the glycoforms tested after incubation with neutrophil elastase, chymotrypsin, or LasB. This suggests that the presence of *N*-glycans in other positions protects against proteolysis. Furthermore, when the human CBG glycosylation mutants were incubated with different amounts of these enzymes, *N*-glycosylation within the RCL appears to protect against proteolysis. This was anticipated because this carbohydrate chain is attached close to the main cleavage sites for these proteases, as mentioned above. Therefore, steric hindrance by the *N*-glycan may reduce the accessibility of proteases to their target sequence in the RCL. In our experiments, we also included a natural human CBG variant in which the RCL carbohydrate chain is missing, i.e., CBG T349A (Simard *et al*. 2015). In individuals with this variant, it is possible that CBG is more susceptible to proteolysis. If so, this may have consequences during infection or inflammation when CBG is targeted by proteases that disrupt its steroid-binding activity (Lin *et al*. 2010, Perogamvros *et al*. 2012).

The greater losses in steroid-binding activity observed when human CBG and its glycosylation mutants were incubated with LasB, when compared to neutrophil elastase or chymotrypsin, suggest that *N*-glycans play an important role in protecting against this bacterial protease. Glycosylation-deficient CBGs were also particularly susceptible to non-specific proteolysis by chymotrypsin and LasB suggesting that these proteases have greater accessibility to CBG when *N*-glycans are missing or altered. However, when human CBG N238 was tested, a molecular weight reduction consistent with cleavage within the RCL appeared initially during incubations with either LasB or chymotrypsin. By contrast, proteolysis by neutrophil elastase appears limited to a single cleavage site under most conditions, and this was most evident when glycosylation-deficient CBGs were tested. Thus, while the carbohydrates attached to CBG may generally preclude cleavage from occurring outside of the RCL, cleavage of the RCL seems to occur preferentially, and secondary sites for proteolysis may only then become more accessible.

In conclusion, *N*-glycans at a similar location in human (N238) and rat (N230) CBGs are necessary for steroid binding, and this may explain why an *N*-glycosylation site is present in the same relative position of all mammalian CBG sequences. Importantly, our experiments provide insight into how quantitative and qualitative differences in *N*-glycosylation influence CBG steroid-binding activity and susceptibility to proteolysis. Our results also imply that *N*-glycosylation acts globally to limit susceptibility to proteolysis and/or restrict cleavage to functionally relevant sites within the RCL. The possibility that specific *N*-glycans associated with CBG influence its function and recognition by other proteins has implications in terms of the actions of CBG during infectious and inflammatory diseases or its detection by antibodies in immunoassays.

### Declaration of interest

The authors declare that there is no conflict of interest that could be perceived as prejudicing the impartiality of the research reported.

### Funding

This work was supported by an operating grant (MOP-111102) from the Canadian Institutes of Health Research (G L H), a Canada Research Chair in Reproductive Health (G L H), and a postdoctoral fellowship from the Fonds de Recherche du Québec en Santé and the Michael Smith Foundation for Health Research (M S).

### Author contribution statement

Conceived experimental design and hypotheses: M S and G L H; performed experiments: M S and C U; analyzed data: M S and C U; wrote the paper: M S and G L H.
